# A mixed methods longitudinal case study exploring the impact of a community-based, brief psychological intervention for men experiencing suicidal crisis

**DOI:** 10.1371/journal.pmen.0000024

**Published:** 2025-04-08

**Authors:** Claire Anne Hanlon, Jennifer Chopra, Jane Boland, David McIlroy, Helen Poole, Pooja Saini

**Affiliations:** 1 School of Psychology, Liverpool John Moores University, Liverpool, United Kingdom; 2 James’ Place, Liverpool, United Kingdom; 3 School of Psychology, Liverpool John Moores University, Liverpool, United Kingdom; PLOS: Public Library of Science, UNITED KINGDOM OF GREAT BRITAIN AND NORTHERN IRELAND

## Abstract

Suicide is a leading cause of death among men globally, highlighting the need for acceptable and effective suicide prevention. This study explored perceptions of the short- and long-term outcomes and acceptability of the James’ Place Model (JPM), a therapeutic intervention delivered within a community-setting for men experiencing suicidal crisis. Also, factors influencing engagement of suicidal men in research were explored. A mixed methods longitudinal case study design was used. Quantitative data was collated through baseline, 3- and 6-month follow up questionnaires distributed to 28 men receiving the JPM. Measures of resilience, hope, generalised self-efficacy, self-compassion, loneliness, perceived social support, entrapment, and the 10-item clinical outcomes in routine evaluation measure were taken, and merged with routine service data. Two semi-structured interviews informed development of case studies exploring men’s perceived acceptabilityand short- and long-term effectiveness of the JPM, and factors relating to suicide research engagement. Descriptive analyses showed mean total scores of entrapment and self-compassion decreased and increased at 3-month follow-up respectively. Mean total scores of entrapment further decreased at 6-month follow-up, while mean scores of self-compassion remained similar to 3-month follow-up. Case studies highlight the perceived acceptability, and short- and long-term outcomes of the JPM suggesting use of the lay your cards on the table component help men to articulate the drivers of their suicidality. Men also discussed continued application of strategies developed during receipt of the JPM long-term including safety planning. The JPM is perceived as acceptable among men experiencing suicidal crisis and future work should seek to determine whether its short-term effectiveness is sustained long-term.

## Introduction

Men are disproportionately at greater risk of suicide than women [1,2]. Office of national statistic (ONS) [3] figures show men accounted for three quarters (4129 deaths) of all suicide deaths (5583 deaths) in England and Wales in 2021. Risk factors associated with suicide uncover a complex interplay of diverse biopsychosocial and behavioural factors [4–7]. Richardson et al., (2021) found sixty-eight risk factors associated with suicide behaviours (including attempts and death) among men [6]. These included sociodemographic characteristics (e.g., marital status, low education); physical health and illness (e.g., smoking, diabetes); mental health problems and psychiatric illness (e.g., anxiety and depression); psychological factors - personality and individual differences (e.g., poor emotional control and aggression); negative life events/trauma (e.g., bereavement and adverse childhood experiences); and characteristics of suicidal behaviour (e.g., history of suicide attempts) [6]. Research has advanced understanding of which factors potentially may drive suicidal thoughts, feelings, and behaviours among men. However, this also highlights the challenge of creating effective suicide prevention approaches that adequately meet men’s needs given the diversity of risk factors [6].

Men are typically portrayed as poor help-seekers who endure greater distress before seeking support for mental health difficulties [8–11]. Adherence to dominant masculine norms, including stoicism and self-reliance, are attributed within the research for undermining men’s expressions of vulnerability and emotion, as well as for their reluctance towards help-seeking [12–16]. However, research is growing which shows that men do seek help but in ways inconsistent with conventional help-seeking behaviours [15–18]. Vickery [16] showed maintaining autonomy in disclosure of emotional distress to professionals who acknowledged the significance of this distress allowed men to reconstruct an alternative masculine ideal conducive to help-seeking and disclosure of emotions. This highlights how content of disclosure and context in which it occurs may interact to influence when men do and do not talk, and what they feel able to disclose [17]. For example, maintaining silence and non-disclosure of suicide may allow men to avoid stigma, but reaffirm masculine norms such as stoicism and control [17]. Findings such as these demonstrate men are willing to seek help and discuss mental health problems, including suicide, when service provision and delivery conditions are suited to their needs and preferences [17,19–21].

Research examining men’s perspectives and experiences of mental health services has shown that men prefer solution-focussed approaches that extend beyond just talking, whereby symptoms and coping strategies are explored [21–23]. Moreover, informal, community-based suicide prevention settings perceived as trustful and which allow reframing of help-seeking to suit masculine norms are preferred [19,24,25]. In response to such evidence, there are growing calls for tailored men friendly suicide prevention services that consider the role of masculinity in the development of, and recovery from, suicidality to improve acceptability and accessibility, and outcomes among men experiencing suicidal crisis [26–28]. However, clinical population-based research examining how suicide risk is managed has dominated, with less attention on community-based suicide prevention [28].

In considering the needs of men experiencing suicidal crisis, the James’ Place (JP) suicide prevention service has been developed. JP is a community-based suicide prevention service for men experiencing suicidal crisis [29]. Therapists are trained to deliver the James’ Place Model (JPM), a brief therapeutic psychological intervention informed by three theories of suicide: the interpersonal theory of suicide [30,31], the collaborative assessment and management of suicidality (CAMS) [32], and the integrated motivational volitional theory of suicide (IMV) [33,34]. The interpersonal theory of suicide describes suicidality as arising from thwarted belonginess, perceived burdensomeness and acquired capability for suicide action [31]. In contrast, the CAMS framework emphasises collaboration between the therapist and individual to understand, assess and treat risk factors and drivers contributing to their suicidality which is guided by the Suicide Status Form [32]. Within the CAMS framework, person-centred and problem-focussed approaches are used to redress suicidal risk factors and drivers [32]. The IMV explains the translation of suicidal thoughts and feelings to behaviours using a tripartite ideation-to-action framework in which feelings of being trapped (entrapment) emerge from inescapable feelings of defeat/humiliation [33,34]. Defeat/humiliation appraisals are triggered by background factors including acute life events and stressors such as relationship breakdown in the pre-motivational phase of the IMV [33,34]. Each theory shares similarities of co-production of effective suicide prevention strategies, safety planning, and equipping the individual to manage their suicidal distress. Also, therapists at JP offer a range of therapeutic approaches and focus upon reducing men’s suicidal distress while improving coping and resilience, consistent with the CAMS approach [32].

Focus of sessions is broadly structured into three components delivered across three sessions each (nine sessions in total) corresponding to safety planning and risk management, delivery of brief psychological interventions (e.g., behavioural activation, sleep hygiene), and relapse prevention involving in-depth safety planning and reflection of progress through the clinical journey. Throughout the clinical journey, the lay your cards on the table (LYCT) component of the JPM is delivered. This novel aspect of the JPM comprises of four stacks of cards which resemble playing cards. Each card within different sets describes either an emotion (e.g., sad, hopelessness), physical sensation (butterflies, dizziness), situation (e.g., someone is bullying me), life event (e.g., end of a significant relationship) or coping approach (final set of cards only – e.g., walk 6000 steps; listen to some music). *What’s happening now* and *how did I get here* cards are delivered during the first three sessions. Next, *keeping the problem going* cards are administered during sessions four to six. The final set of cards, *how can I get through this*, are delivered during the last three sessions. The purpose of the cards is to prompt discussion around specific issues and correspond to specific stages of the JPM as described. More detailed information about the JP service and JPM are available elsewhere [29,35–37].

Evaluation is a key facet of JP practice, and outcomes are routinely assessed using the clinical outcomes in routine evaluation-10 (CORE10) and entrapment short form (E-SF) questionnaires [38,39]. Evaluation studies have shown that the JPM is effective in supporting men experiencing suicidal crisis in the short-term [35–37]. Most recently, evaluation of year three service data showed a significant reduction upon discharge from JP in clinical outcomes in routine evaluation-10 (CORE10) and entrapment (E-SF) scores, which measure psychological distress and an individual’s perceptions of feeling trapped internally by their thoughts and feelings, and/or by external situations respectively [37,38]. While evidence supports the short-term effectiveness  of the JPM, less is known about its effectiveness  post-intervention and whether significant reduction in psychological distress and entrapment is sustained post-discharge from the service. This study therefore aims to explore the short- and long-term outcomes of the JPM using data collected at baseline, 3- and 6-month follow up, by addressing the following research questions:

1. Does the JPM reduce psychological distress, entrapment, and loneliness among men experiencing suicidal crisis?
2. Does the JPM improve hope, generalised self-efficacy, perceived social support, resilience and self-compassion among men experiencing suicidal crisis?
3. What are the experiences of men who have received the JPM during and after intervention delivery?
4. What factors influence the perceived acceptability and feasibility of conducting long-term research among men who have received the JPM for suicidal crisis?

## Materials and methods

### Ethics statement

Ethical approval was given by Liverpool John Moores University research ethics committee (Ref:20/NSP/043). Written consent was gained from men using the service at their initial welcome assessment. Written consent was also obtained from men who took part in the semi-structured interviews.

### Design

A mixed methods longitudinal case study approach was used. Questionnaire data was merged with data routinely collected by the JP service. The qualitative phase included two semi-structured interviews with men who had received the JPM, and data from these were developed into two case studies.

### Participants

Purposive sampling was used to recruit male participants who were in receipt of the JPM for suicidal crisis (see Hanlon et al., [29] for further details). Twenty-eight men completed baseline questionnaires. Each was emailed an online follow-up questionnaire at 3- and 6-month follow-up. Two men who completed baseline questionnaires also took part in a semi-structured interview.

### Measures

Total mean scores of the following measures comprised baseline, 3- and 6-month questionnaires. Baseline measures of entrapment (E-SF) and clinical outcomes in routine evaluation-10 (CORE10) were obtained from JP routinely collected data. The decision to omit entrapment (E-SF) and clinical outcomes in routine evaluation-10 (CORE10) measures at baseline was taken by the request of the JP service to avoid over-burdening men since these are taken upon acceptance to the service.

*Demographic characteristics:* Information including age, relationship status, preferred mode of delivery of the JPM (e.g., in-person, online, telephone) and alternative support services men would have sought support from if they had not approached JP (e.g., A&E, GP) were recorded.

#### Measures to Explore the Reduction of Psychological Distress, Entrapment and Loneliness.

*Clinical Outcomes in Routine Evaluation-10 (CORE10):* The clinical outcomes in routine evaluation-10 (CORE10) [39] assessed psychological distress and includes 10-items (e.g., “*I made plans to end my life*” and “*unwanted images or memories have been distressing me*”). The clinical outcomes in routine evaluation-10 (CORE10) utilises a 5-point Likert scale ranging from 0 (*“not at all”*) and 4 (*“most or all of the time”*) and respondent scores give a total score ranging from 0 to 40. Higher clinical outcomes in routine evaluation-10 (CORE10) scores indicate higher levels of psychological distress. Clinical outcomes in routine evaluation-10 (CORE10) scores of less than 10 corresponds to the non-clinical range; 11 to 14 mild psychological distress; 15 to 19 moderate psychological distress; 20 to 24 moderate-to-severe psychological distress; 25 or above severe psychological distress. A total score of 11 or above represents the clinically significant range.

*Entrapment:* Entrapment short form scale (E-SF) [38] measured four items relating to external entrapment (e.g*., “I am in a situation I feel trapped in”*) and *internal entrapment (e.g., “I want to get away from myself”)*. As a self-report measure respondents are asked to endorse their response along a 5-point Likert scale ranging from *“not at all like me” (0)* to *“extremely like me” (4)* providing a potential range of total scores from 0 to 16. An overall score of entrapment is calculated by adding each item score with higher total scores indicative of higher levels of entrapment.

Both the clinical outcomes in routine evaluation-10 (CORE10) and entrapment short-form scale (E-SF) correspond to routine outcome measures used by JP and were included in each follow-up questionnaire. It is not possible to calculate a reliability score for either the clinical outcomes in routine evaluation-10 (CORE10) and/or entrapment short-form scale (E-SF) as JP record total scores rather than individual item scores on these scales for each man. Note, from the 1^st^ September 2020 the entrapment short-form scale (E-SF) was introduced as a routine measure of internal and external entrapment at JP.

*Loneliness:* The revised UCLA loneliness scale-8 (ULS8) [40] measured loneliness. Adapted from the revised UCLA-20 loneliness scale [41], eight items comprise the UCLA loneliness scale-8 (ULS8) [40]. A 3-point Likert scale was used in the present study with values ranging from 1 to 3 representing *“hardly ever or never”* and *“often”* respectively. Two items were reverse scored (*“I am an outgoing person”* and *“I can find companionship when I want it”*) and each item score summed to create a total score. Potential total scores range from 8 to 24, with higher scores indicative of greater loneliness. A Cronbach alpha coefficient score of.55 indicates low internal consistency for this scale in this study.

#### Measures to Explore the Improvement of Resilience, Hope, Generalised Self-Efficacy, Self-Compassion and Perceived Social Support.

*Resilience:* The six-item brief resilience scale (BRS) [42] was used to assess resilience. Items (e.g., “*I tend to bounce back”* and *“I usually come through difficult times with little trouble”*) are assessed along a 5-point Likert scale ranging from strongly disagree to strongly agree. Items comprising the brief resilience scale (BRS) are either positively (3 items) or negatively worded (3 items). Brief resilience scale (BRS) scores were acquired by summing all 6-item scores and dividing the total sum of scores by six to give a total mean score ranging from 1-6, with higher total mean scores indicative of higher perceived resilience. Good reliability was achieved for this scale in the present study (α=.81).

*Hope:* The 12-item adult hope scale (AHS) [43] was used in the present study. The adult hope scale (AHS) is comprised of two subscales including agency (4-items) and pathways (4-items) which correspond to goal-orientated energy and planning to accomplish goals respectively. Example items include *“My past experiences have prepared me well for my future”* (agency) and *“I can think of many ways to get out of a jam”* (pathways). The remaining four items are filler items (*“I feel tired most of the time”, “I am easily downed in an argument”, “I worry about my health”* and *“My past experiences have prepared me well for my future”*) which were excluded from the analyses. Individual scores are ranked along an 8-point Likert scale ranging from *“definitely false”* to *“definitely true”* corresponding to a score ranging from 1 to 8 respectively. Scores can be assessed at the subscale level or as a total score. In the present study, agency and pathways items were summed to give a total score ranging from 8 to 64, with higher scores reflecting higher hope. The scale accrued moderate reliability in the present study (α=.67).

*Generalised self-efficacy:* The 10-item general self-efficacy scale (GSE) [44] was used to assess generalised self-efficacy. Scale items include *“I am confident that I could deal efficiently with unexpected events”* and *“If I am in trouble, I can usually think of a solution”* which are measured along a 4-point Likert scale ranging from one to four representing *“not at all true”* and *“exactly true”* respectively. Total generalised self-efficacy (GSE) scores range from ten to forty, with higher scores indicative of higher generalised self-efficacy. The high alpha value (α=.92) shows good internal consistency was achieved for this scale.

*Self-compassion:* Neff’s 26-item self-compassion (SC) scale [45] consists of six subscales: self-kindness, self-judgement, common humanity, isolation, mindfulness and over identification. Items (e.g., “*I’m tolerant of my own flaws and inadequacies”* and *“When I fail at something important to me, I try to keep things in perspective”*) are assessed along a 5-point Likert scale ranging from *“almost never”* (1) to *“almost always”* (5). To ascertain a total score of self-compassion, self-judgment, isolation, and over identification subscales were reverse scored. Mean scores of each subscale and then a total mean score of all six subscales was calculated. Higher total mean scores were indicative of higher self-compassion. A high Cronbach alpha coefficient value (α=.81) shows this scale achieved high reliability in this study.

*Perceived social support:* The multidimensional scale of perceived social support (MSPSS) [46] consists of 12-items with responses indicated using a 7-point Likert scale ranging from *“very strongly disagree”* (1) to *“very strongly agree”* (7). Three subscales of family, friends and significant others comprise the scale, with mean total score representative of the perceived adequacy of social support from these sources. In the present study, a total mean score was calculated by summing each item response across all 12-items and dividing by 12. Higher mean total scores indicate higher levels of perceived social support. The scale achieved high reliability in the present study (α=.90).

### Procedure

Men accepted into JP between 29^th^ December 2020 and 11^th^ April 2022 were invited to complete a questionnaire at baseline. Twenty-eight men completed a baseline questionnaire, with seventeen agreeing to be sent follow-up questionnaires. Of the men sent questionnaires at follow-up, twelve and three men completed 3- and 6-month follow-up questionnaires respectively.

Men who had completed the JPM and a baseline questionnaire, who had agreed to being contacted by a researcher for follow-up, were invited to participate in an interview. Ten men agreed to be followed up for interview. Seven men were excluded because they had either re-engaged with the JP service (n = 4), were deceased (n = 1) or for other reasons (n = 2). The purpose of interviews was to explore men’s views on how effective they perceived the JPM was in supporting them through suicidal crisis and in the period post-crisis. Response rate to the invitation to take part was poor (n = 2; 20%). Subsequently, two men agreed to participate in an individual semi-structured interview. Written consent was obtained from participants prior to them being interviewed, and interviews took place at JP Liverpool and over the telephone on the 21^st^ December 2022 and 1^st^ of February 2023 respectively.

### Data analysis

Routine clinical information compiled by JP was accessed and merged with baseline and 3- and 6-month follow-up data, and descriptive analyses conducted. Routine data includes sociodemographic details of men (age, ethnicity), and precipitating and psychological factors experienced by men upon entry to JP, and clinical outcomes in routine evaluation-10 (CORE10) and entrapment (E-SF) data.

Semi-structured interviews were audio recorded using a Dictaphone and transcribed verbatim using a transcription service, generating 66 minutes of interview data. Resultant data was analysed using Richie and Spencer’s [47] five stages of thematic framework analysis to explore participants perceptions of the JPM and their experiences of participating in questionnaire studies in suicide prevention related research.

## Results

### Sample characteristics

Between 1^st^ December 2020 and 15^th^ April 2022, James’ Place received 742 referrals from emergency departments, primary care, Universities, or self-referrals. Of 391 men offered a welcome assessment, 341 went on to engage in therapy at JP. For those who did not attend their welcome assessment, the reason was usually because men were no longer feeling suicidal or there was no response when the service attempted to contact men to arrange the welcome assessment. During this period, one man was referred on to an alternative service. The specific service they were referred to and for what reason is unknown as this information was not recorded in the service data. Of 341 men who attended during this period, twenty-eight completed a baseline questionnaire. Questionnaire data for each man who took part in this study (*N*=28) was merged with routine data collated by JP using each man’s unique identification case number.

Mean age of participants in the study was 43 years (range 22-66 years; SD=11.45 years). The mean number of sessions attended by men was seven (SD=2.87). Typically, men attending JP are offered up to 9 sessions of therapy with a specialised suicide prevention therapist. This number of sessions is comparable to the number of sessions typically attended by men accessing JP as reported in JP evaluation reports (i.e., range 6-7 sessions) [35–37]. Follow-up questionnaires were completed by men from 12^th^ February 2021 to 14^th^ March 2022. Significant attrition was observed at follow-up with thirteen questionnaires completed at 3-month follow-up and three questionnaires completed at 6-month follow-up, representing an attrition rate of 54% and 89% respectively.

Table 1 shows the sample demographics (*N=*28) and service data demographics (*N*=742) for the period from 1^st^ December 2020 to 15^th^ April 2022.

**Table 1:** Demographic Characteristics of Study Sample and James’ Place Service Data from 1^st^ December 2020 to 15^th^ April 2022.

Variable	*N* (%) Study Sample (*N*=28)	*N (%)* Service Data^*^ (*N*=742)
*Ethnicity*		
White British	24 (85.7)	531 (71.6)
Other ethnicity	0	144 (19.4)
Not specified	4 (14.3)	67 (9)
*Relationship status*		
Single	9 (32.1)	408 (55.0)
Married	10 (35.7)	85 (11.5)
In a relationship	5 (17.9)	127 (17.1)
Divorced	1 (3.6)	8 (1.1)
Separated	0	31 (4.2)
Widowed	0	5 (.7)
Not specified	3 (10.7)	78 (10.4)
*Sexual orientation*		
Heterosexual	13 (46.4)	348 (46.9)
Homosexual	0	44 (5.9)
Bisexual	1 (3.6)	6 (.8)
Not specified	14 (50)	344 (46.4)
*Employment status*		
Employed	15 (53.6)	292 (39.4)
Unemployed	8 (28.6)	266 (35.8)
Students	0	78 (10.5)
Carer	0	4 (.5)
Retired	2 (7.1)	10 (1.3)
Not specified	3 (10.7)	92 (12.5)
*Postcode deprivation*		
Most deprived	23 (82.1)	575 (77.5)
Least deprived	5 (17.9)	121 (16.3)
Not specified	0	46 (6.2)

*Service data for men referred to JP during the period from 1^st^ December 2020 to 15^th^ April 2022.

All men who provided ethnicity data identified as white British (n=24; 85.7%). Most men reported that they were employed (n=15; 53.6%) and reside in the most deprived areas as indicated by Index of Multiple Deprivation (IMD) post-code scores (n=23; 82.1%). Relationship status varied among participants, but most men were married (36%) or single (32%). Of fourteen men who provided details about their sexuality, thirteen identified as heterosexual. Some similarities and differences were seen between sample demographic data of men who participated in the present study compared to demographic data of men referred to JP during the period the study period. Of 742 men referred to JP during the study period (i.e., from 1^st^ December 2020 to 15^th^ April 2022), most were White British (n=531; 71.6%), employed (n=292; 39.4%), reside in the most deprived areas (n=575; 77.5%) and are heterosexual (n=348; 46.9%). However, in contrast to the study sample most men referred to JP during the study period were single (n=408; 55%).

### Psychological Profile of Men

Men attributed several factors to precipitating their suicidal crisis upon entry to JP shown in Table 2.

**Table 2:** Precipitating Factors Pre-Baseline (upon entry to James’ Place) Attributed to Suicidal Crisis (*N*=28).

Precipitating factor	*N* (%) Recorded by service^*^
Relationship problems	13 (46.4)
Work	8 (28.6)
Health problems	7 (25)
Bereavement	6 (21.4)
Financial issues	5 (17.9)
Covid-related issues	4 (14.3)
Victim of past abuse trauma	3 (10.7)
Substance/alcohol misuse	3 (10.7)
Legal problems	2 (7.1)
Family problems	2 (7.1)
Carer	2 (7.1)
Sexuality	2 (7.1)
Perpetrator of crime	1 (3.6)
University	1 (3.6)
Housing issues	1 (3.6)
Other	0

*Note: figures reflect collapsed variables, therefore it is feasible an individual’s data has been recorded more than once.

The two most prevalent precipitating factors for men in the study sample were relationship problems (n=13; 46.4%) and work (n=8; 28.6%). This highlights the important role of social and relational proximal factors in contributing to the men’s suicidal distress. In contrast, the least prevalent precipitating issue was the other category (n=0) which includes factors such as bullying and asylum-related issues.

Table 3 shows the prevalence of psychological factors reported by men upon acceptance to JP.

**Table 3:** Psychological Factors Pre-Baseline (upon entry to JP) Attributed to Suicidal Crisis.

Psychological variable	N (%) Recorded by service	N (%) Missing data
Entrapment (n=26)	21 (75)	2 (7.1)
Rumination (n=26)	19 (67.9)	2 (7.1)
Social support (n=25)	19 (67.9)	3 (10.7)
Burdensomeness (n=25)	18 (64.3)	3 (10.7)
Thwarted belongingness (n=26)	16 (57.1)	2 (7.1)
Past suicide attempt or self-harm (n=26)	16 (57.1)	2 (7.1)
Defeat (n=26)	15 (53.6)	2 (7.1)
Social problem solving (n=26)	13 (46.4)	2 (7.1)
Memory biases (n=26)	13 (46.4)	2 (7.1)
Not engaging in new goals (n=26)	13 (46.4)	2 (7.1)
Impulsivity (n=26)	13 (46.4)	2 (7.1)
Imagery of death by suicide (n=26)	12 (42.9)	2 (7.1)
Humiliation (n=25)	10 (35.7)	3 (10.7)
Absence of positive future thinking (n=26)	10 (35.7)	2 (7.1)
Suicide plan (n=26)	9 (32.1)	2 (7.1)
Resilience (n=26)	9 (32.1)	2 (7.1)
Exposure to suicide (n=25)	8 (28.6)	3 (10.7)
Pain sensitivity tolerance (n=25)	7 (25)	3 (10.7)
Coping (n=25)	7 (25)	3 (10.7)
Fearlessness of death (n=25)	5 (17.9)	3 (10.7)
Unrealistic goals (n=25)	4 (14.3)	3 (10.7)
Attitudes (n=25)	3 (10.7)	3 (10.7)
Social norms (n=25)	1 (3.6)	3 (10.7)

Entrapment was the most frequently reported psychological factor by men (n=21; 75%) suggesting most men were experiencing inescapable feelings of being trapped by external situations and by their own thoughts and feelings. In contrast, social norms were the least frequently reported psychological factor by men (n=1; 3.6%) suggesting these were less important in relation to the experience of suicidal crisis among men who took part in the present study.

### Questionnaire Completion Rates

Table 4 shows the number of participants who completed study questionnaires at baseline, 3- and 6-month follow-up. Significant attrition rates at 3- and 6-month follow-ups occurred across the study follow-up period (reported earlier).

**Table 4:** Completion rates of baseline, 3- and 6-month follow-up questionnaires.

Variable Measure	Baseline (N=28)	3-month follow-up (N=13)	6-month follow-up (N=3)
Brief Resilience Scale (BRS)	n = 28	n = 13	n = 3
Adult Hope Scale (AHS)	n = 27	n = 13	n = 3
Generalised self-efficacy Scale (GSE)	n = 27	n = 13	n = 3
UCLA Loneliness Scale-8 (ULS8)	n = 27	n = 10	n = 3
Self-Compassion Scale (SC)	n = 27	n = 10	n = 3
Multi-dimensional Scale of Perceived Social Support (MSPSS)	n = 28	n = 10	n = 3
Clinical Outcomes in Routine Evaluation-10 scale (CORE10)	n = 27	n = 12	n = 3
Entrapment Short Form Scale (E-SF)	n = 27	n = 12	n = 3

### Baseline and Follow-Up Questionnaire Results

Table 5 shows the mean total scores and standard deviations for the baseline and 3- and 6-month follow-up scores for each psychological measure within the questionnaire. It is not possible to accurately compare baseline data with follow-up data due to the small sample size and amount of attrition.

**Table 5:** Mean and Standard Deviations of Baseline, 3- and 6-month Follow-up Questionnaires (*N*=28).

Variable	Completion rates (N)	Mean (SD)	Min. score	Max. score
*Brief Resilience Scale(BRS)*				
Baseline	28	2.24 (.79)	1	4.17
3-month follow-up	13	2.87 (1.26)	1	5.33
6-month follow-up	3	2.33 (1.53)	1	4
*Adult Hope Scale (AHS)*				
Baseline	27	55.78 (10.63)	30	76
3-month follow-up	13	55.62 (11.84)	36	74
6-month follow-up	3	45 (10)	35	55
*Generalised Self-Efficacy Scale (GSE)*				
Baseline	27	23.26 (5.76)	11	34
3-month follow-up	13	24.69 (7.23)	10	34
6-month follow-up	3	20.67 (9.71)	10	29
*UCLA Loneliness Scale-8 (ULS-8)*				
Baseline	27	18.30 (2.71)	12	24
3-month follow-up	10	13.40 (7.47)	4	24
6-month follow-up	3	16.67 (6.81)	9	22
*Self-Compassion Scale (SC)*				
Baseline	27	1.95 (.61)	1	3.08
3-month follow-up	10	3.14 (.54)	2.35	4.36
6-month follow-up	3	3.28 (.30)	3.07	3.63
*Multi-dimensional Scale of Perceived Social Support (MSPSS)*				
Baseline	28	3.63 (1.25)	1	5.83
3-month follow-up	10	4.26 (1.43)	2.17	6.08
6-month follow-up	3	3.72 (2.47)	1.42	6.33
*Clinical Outcomes in Routine Evalution-10 Scale (CORE10)*				
Baseline	27	30.70 (6.01)	15	38
3-month follow-up	12	37.75 (5.10)	29	45
6-month follow-up	3	37.33 (11.37)	28	50
*Entrapment Short-Form Scale (E-SF)*				
Baseline	27	14.15 (2.44)	8	16
3-month follow-up	12	12.08 (3.92)	4	19
6-month follow-up	3	11.67 (8.02)	4	20

From baseline to 3-month follow-up, mean total scores of generalised self-efficacy (GSE), self-compassion (SC) and perceived social support (MSPSS) increased, loneliness (ULS-8) decreased, while hope (ALS) and resilience (BRS) remained similar to baseline mean total scores. In contrast, mean total scores of hope (ALS), generalised self-efficacy (GSE), and perceived social support (MSPSS) decreased and loneliness (ULS-8) increased at 6-month follow-up, while resilience (BRS) and self-compassion (SC) remained similar to 3-month follow-up mean total scores. However, self-compassion (SC) recorded a higher mean total score at 6-month follow-up than at baseline. Further, mean total clinical outcomes in routine evaluation-10 (CORE10) scores increased from baseline to 3-month follow-up and remained comparable at 3- and 6-month follow-up. While mean total entrapment (E-SF) scores decreased from baseline to 3-month follow-up and from 3- to 6-month follow-up also.

### Descriptive case study findings

**Fig 1:**
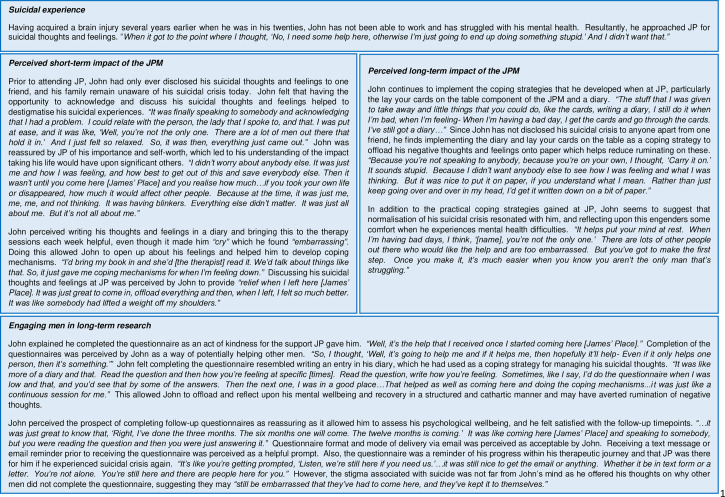
Case study 1: John (56 years old).

**Fig 2:**
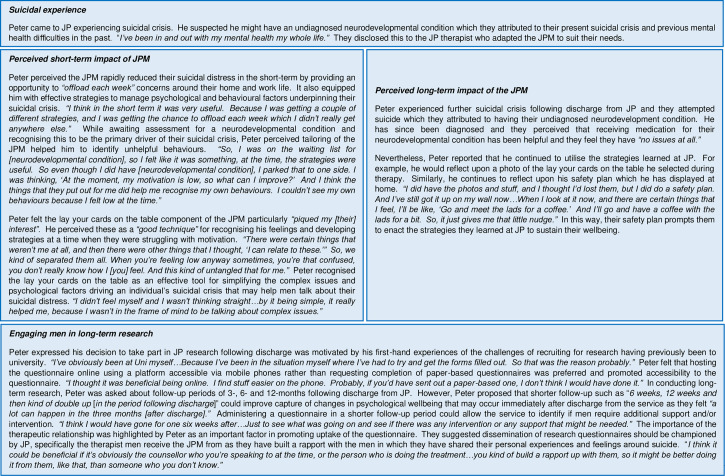
Case study 2: Peter (35 years old).

Three themes were developed to capture men’s experiences of the short-term and long-term impact of the JPM following discharge from the service and the feasibility and acceptability of conducting long-term research with men following suicidal crisis and are reported in figures 1 and 2.

## Discussion

### Summary of findings

This study aimed to explore the short- and long-term outcomes of the JPM among men experiencing suicidal crisis. More specifically, whether the JPM reduced key risk factors associated with suicide including psychological distress (clinical outcomes in routine evaluation-10), entrapment (E-SF) and loneliness (ULS8) and increased potentially protective factors of resilience (BRS), hope (ALS), generalised self-efficacy (GSE), perceived social support (MSPSS) and self-efficacy (SC) at baseline and 3- and 6-month follow up periods. Descriptive case studies further illustrate men’s experiences of the JPM during and post-intervention, and their perceived acceptability and feasibility of conducting long-term research among men following suicidal crisis which are captured by three themes. The first, *perceived short-term impact of the JPM* which describes how men interacted with the model and the perceived impact this had upon reducing suicidal crisis. Second, the *perceived long-term impact of the JPM* which highlights how men continue to implement strategies to avert relapse once they had completed the intervention. The final theme, *engaging men in long-term research* highlights men’s motivations and reasons for participating in this study and their views on how researchers can better engage men who have experienced suicidal crisis in future suicide-related research.

Several precipitating factors and psychological factors were endorsed by men upon receiving a JP welcome assessment, of which relationship breakdown and work, and entrapment were reported respectively. Mean total scores of loneliness (ULS-8) and entrapment (E-SF) decreased from baseline to 3-month follow-up suggesting men who had received the JPM felt less isolated and trapped within their situation and thoughts and feelings of suicide. Mean total scores of hope (ALS) and resilience (BRS) remained similar between baseline and at 3-month follow up. However, mean total scores of generalised self-efficacy (GSE), perceived social support (MSPSS), self-compassion (SC) and clinical outcomes in routine evaluation-10 (CORE10) increased between baseline and 3-follow-up. This suggests improved self-efficacious beliefs, social support, and self-compassion, yet increased psychological distress among men after they have completed the JPM. Hope (ALS), generalised self-efficacy (GSE), perceived social support (MSPSS) and entrapment (E-SF) mean total scores decreased at 6-month follow-up compared to 3-month follow-up, while loneliness (ULS-8) mean total scores increased. However, resilience (BRS), self-compassion (SC) and clinical outcomes in routine evaluation-10 (CORE10) scores remained similar at 3-month follow-up compared to 6-month follow-up. This suggests that men experienced a decline across several psychological variables including those associated with improved coping at 6-month follow up indicating men felt less hope, social support, and generalised self-efficacy yet felt less trapped within their situation and feelings, and felt lonelier. However, levels of the ability to recover from stress, self-compassion and psychological distress were maintained at 6-month follow-up. Thus, highlighting the complexity of suicidal experiences and recovery following intervention.

Poor participant uptake and high attrition rates at follow-up prevented multivariate statistical analysis of questionnaire results, therefore it is not possible to establish causal relationships. Despite the somewhat varied and contradictory findings, descriptive case study findings suggest that the JPM may be perceived as an acceptable therapeutic approach among suicidal men.

### Interpretation of results

Frequency of the psychological factor of entrapment and precipitating factors of relationship breakdown and work-related issues in the present study is consistent with the IMV theory of suicide in explaining the development of suicide ideation and behaviours [33,34]. Additional psychological variables of thwarted belonginess and perceived burdensomeness consistent with the interpersonal theory of suicide, were identified by over half of men pre-baseline (57% and 64% respectively) [30,31]. These findings support integration of the assessment of entrapment, thwarted belonginess and perceived burdensomeness in the routine clinical assessment of suicidality for men. However, given that men endorsed several additional precipitating and psychological factors, this highlights the breadth and uniqueness of suicidality among men and the need to explore these within a therapeutic framework to understand how these factors contribute to suicidal crisis.

Relationship breakdown and work-related issues were frequently reported precipitating factors for suicide among men which aligns with previous research [6,48]. It was not possible to define the nature of relationship breakdown (e.g., divorce, separation) or work issues among the men (e.g., stress, job loss, job insecurity) as this detail had not been recorded in the service data provided. However, the separation period during relationship breakdown presents a particularly vulnerable period for men compared to women with an increased risk of suicide four times greater than non-separated men [49,50]. Similarly, Mughal et al., [51] in examining sociodemographic characteristics and antecedents of middle-aged men whose final GP consultation occurred 3-months prior to their death by suicide found 29% (n=30 of 105) and 33% (n=35 of 105) of middle-aged men reported experiencing recent work-related problems and were unemployed respectively.

Past research has attributed men’s’ vulnerability to suicide associated with relationship breakdown and work-related issues to dominant masculine norms [48,52,53]. Accordingly, feelings of shame may arise from undermined traditional masculine ideals such as being the provider [52,54]. Culmination of men’s’ propensity for restrictive emotional expression, overreliance upon independence and intimate partner emotional and social support limits adaptive help-seeking behaviours [48,53]. Subsequently, men perceive support for their distress as inaccessible and unavailable [48]. Nevertheless, the present findings demonstrate some men do seek and engage with psychological support if the clinical setting is suitable. Thus, adding further support for the need for suicide prevention approaches that are sensitive towards men’s help-seeking needs and preferences [21].

It was not possible to test the effectiveness of the JPM due to poor uptake and follow-up rates. However, men perceived that the JPM was effective in reducing their suicidal crisis. Men described a pattern of avoiding help-seeking until they reached a critical moment where they were no longer able to cope, and for one man, they rationalised their reaching out for help from JP as necessary for their survival. Displays of self-reliance as reported here can be explained within the context of masculine norms, and the reframing of active help-seeking as a necessity to safeguard survival once men reach their lowest point is consistent with previous research [48,55,27]. Importantly, this emphasises there is a window of opportunity during which engagement with mental health services could interrupt men’s suicidal thinking and intent, provided the intervention content and context is primed to men’s needs [17,18]. It was reported that JP therapists worked with men in a way that allowed them to *“offload”* and normalised their suicidal experiences while collaboratively working with men to support development of coping strategies to manage their suicidal thoughts. Men particularly noted the dynamic nature of the LYCT component of the JPM in helping them to articulate and organise their thoughts and feelings around their suicidal thinking, as well as the solution-focussed approach on developing coping strategies which men reported they continue to use. This highlights the importance of community-based services which promote therapeutic alliance and, through shared decision-making, person-centred and solution-focussed approaches sensitive to the influence of masculinity on suicide risk and help-seeking behaviour [22,27,56].

Case study findings suggest the JPM is feasible and acceptable among men experiencing suicidal crisis. However, it was reported by one man (Peter) that he did go on to attempt suicide some months after being discharged from the JP service. Research has shown that brief psychological interventions for suicide prevention are effective in reducing suicide and suicide attempts, but they do not reduce suicide ideation [57,58]. This underscores the complex nature of suicide and how some men may require additional support once they have completed the JPM and been discharged from the service. In recognition of this, JP has developed referral pathways with a range of local community support services enabling therapists to refer men for additional support for a range of psychosocial issues (e.g., housing, debt management). Additionally, therapists reiterate to men that they can utilise the mentoring service delivered by JP volunteers and be referred back to JP should they experience suicidal crisis again in the future.

Previous studies have shown the JPM significantly reduces suicidal distress among men experiencing suicidal crisis [19,20,24]. It was not possible to test the effectiveness of the JPM in the present study due to the small sample size. However, examination of mean total clinical outcomes in routine evaluation-10 (CORE10) scores at baseline compared to 3- and 6-month follow-up revealed men were experiencing severe psychological distress at 3-month follow-up which remained similar at 6-month follow-up. Indeed, some men who had completed baseline questionnaires (n=4) were found to have re-engaged with JP for support for suicidal crisis. However, most men were found to have not re-engaged with JP. While it is possible men may have sought support elsewhere, it is feasible men could have felt capable of keeping themselves safe during this period by implementing the coping strategies they developed through the JPM (e.g., their safety plan). In recognising the vulnerable period experienced by individuals following suicidal crisis (e.g., Vatne and Nåden [59]), a key feature of the JPM is a focus on preventing reoccurrence of suicidal crisis. Drawing upon the key principles of flexibility and co-production of treatment of the CAMS therapeutic framework, therapists at JP work with men to co-produce coping strategies and a bespoke safety plan, and to develop men’s self-awareness to recognise changing moods and feelings that may progress to suicidality. Research has shown that safety plan interventions and coping strategies that promote internal coping and resilience, particularly distraction or positive activity-oriented strategies (e.g., socialising and keeping busy) and those pitched to the level of risk experienced by the individual, are effective in preventing suicide [55,60,61]. The final phase of the JPM involves therapists working with men to reflect upon their safety plan and coping strategies acquired through their therapeutic journey at JP. This typically involves delivery of the *how can I get through this* set of LYCT which contains cards that encourage men to seek social support (e.g., “talk to a friend”) and activities to distract them from their suicidal thoughts (e.g., “go for a walk”, “listen to music”). Consistent with this, men reported in the case study findings that they frequently reflected upon the coping strategies they learned through the JPM when they recognised a downward turn in their psychological wellbeing. Development of improved coping following completion of the JPM could also explain reduced total mean entrapment scores at 3- and 6-month follow-up. However, this supposition is speculative and further research is needed to examine this.

The findings also suggest further assessment of the role of self-compassion in mitigating suicidality among men is warranted. Follow-up data shows elevated mean total scores of psychological distress (clinical outcomes in routine evaluation-10; CORE-10), unchanged resilience (BRS), and fluctuating hope (ALS), generalised self-efficacy (GSE), loneliness (ULS-8), and perceived social support (MSPSS) scores at 3- and 6-month follow-up compared to baseline. However, mean total scores of entrapment (E-SF) decreased from baseline to 3-month follow-up, and from 3- to 6-month follow-up, while self-compassion (SC) scores increased and remained similar at 3- and 6-month follow-up despite increased psychological distress (clinical outcomes in routine evaluation-10; CORE10 scores). Indeed, closer inspection of the mean total self-compassion (SC) scores shows a very small marginal increase from 3- to 6-month follow-up. This may indicate a potential adaptive role for feelings of self-compassion for mitigating changes in psychological distress during recovery following suicidal distress. While research in relation to self-compassion and suicide is limited, systematic review findings show self-compassion and self-forgiveness are negatively associated with suicide ideation and suicide attempts [62]. Self-compassion extends beyond self-criticism, as a self-compassionate individual possesses self-awareness of their own personal failings and a mindful and non-judgmental awareness of emotionally painful experiences [45,63]. Indeed, case study findings showed John perceived that sharing his suicidality with a JP therapist helped to destigmatise and normalise their suicidal experience (e.g., *“Well, you’re not the only one. There are a lot of men out there that hold it in.”*). It could be speculated that John may have reframed his suicidal experiences with mindful awareness by acknowledging suicidal thoughts and feelings as a common human experience rather than reflective of his own personal failure. However, further research would be required to determine whether the JPM engenders the development of increased self-compassion.

Sample size limitations mean it is not possible to determine the feasibility of conducting long-term research among men who have received the JPM for suicidal crisis. However, men interviewed as part of this study expressed a willingness to be involved in research after they have been discharged from JP which appears to be underpinned by a sense of altruism. Yet, poor uptake and high attrition rates raises the question on how better to improve adherence to large-scale studies in suicide prevention research. The brief nature of the JPM mean men are under the service for a short period of time making it harder for researchers to engage with them. Also, men leaving the service may feel less inclined to revisit their experiences of suicidality once they are discharged so as not to disrupt their recovery [64]. While men did not criticise the questionnaire design and administration methods used in this study, previous research has suggested inclusion of strategies including monetary incentives and ensuring accessibility of research materials could improve retention in mental health-based research [65,66]. Future research aiming to assess the long-term effectiveness of the JPM may seek to co-produce materials with key stakeholders to improve recruitment rates. Furthermore, future research should also seek to understand the mechanisms underpinning change among men who receive the JPM, with self-compassion and entrapment warranting attention, to understand how the JPM is helping men.

### Strengths and limitations

A key strength of this study is the inclusion of men experiencing suicidal crisis who are receiving a clinical intervention in a community-based suicide prevention service setting [19,24,67]. Research typically involves clinical samples in clinical settings, and there is a paucity of research examining suicide prevention within the community. Suicide related studies are often retrospective or involve psychological autopsy which can be susceptible to recall bias and/or, in the case of psychological autopsy, a limited range of prominent risk factors may be captured [68]. Therefore, the study findings shed some light on the type and frequency of precipitating and psychological factors experienced by men in suicidal crisis upon accessing a community-based suicide prevention intervention.

This study is unlikely to have captured all risk factors associated with men’s experience of suicidality given the small sample size in the present study. Further, poor uptake and high attrition rates, as well as the complex nature of suicide, limit generalisability of the results to a wider community-based population of men experiencing suicidal crisis. It should also be noted that although many men may have risk factors associated with suicide, they never go on to think about or attempt suicide. Several methodological limitations should also be considered when interpreting the results of this study. The present study is an observational study therefore it does not facilitate a control group comparison. It is therefore not possible to infer causality of fluctuations of suicide risk for the duration of the study follow-up period. The study results may also be subject to several types of bias including confounding bias due to additional factors exerting an effect upon clinical outcomes in routine evaluation-10 (CORE10) scores (e.g., age), recall bias due to participant recall error, and ascertainment error bias (i.e., confirmation bias) [69,70]. Participants involved in this study were self-selecting, and this may have been influenced by the degree of suicidal distress experienced by each man [71]. For example, men who felt more overwhelmed by their suicidality may have opted not to participate [71]. Further, as participants indicated their ethnicity as white British, the results may not be representative of men across a wider ethnic demographic. Future longitudinal research should strive to overcome the recruitment issues identified to test whether the positive effects of the JPM reported to date are sustained.

## Conclusion

More research is required to understand the intersection between men’s suicidality, pathways to help-seeking, engagement with services and the efficacy of services. The present study supports intervention delivery that is tailored to men’s individual needs and promotes dynamic interaction with specialised suicide prevention therapists. Earlier findings point to the positive outcomes and value of the JPM, however practical challenges of research engagement must be considered to achieve sufficient longitudinal data to test the long-term effects of the JPM.
